# Regarding the interpretation of creatinine clearance changes following loop diuretics in the intensive care unit

**DOI:** 10.1080/0886022X.2026.2696620

**Published:** 2026-07-06

**Authors:** Muammer Avcı, Özgür Timuçin Kutlu

**Affiliations:** a Department of Nephrology, Isparta City Hospital, Republic of Turkey Ministry of Health, Isparta, Turkey; b Department of Internal Medicine, Isparta City Hospital, Republic of Turkey Ministry of Health, Isparta, Turkey

We have read with great interest the manuscript by Kwok M. Ho et al. titled *Association between the initiation of loop diuretics and changes in 2-h creatinine clearance in critically ill patients* [1], where 31 critically ill patients received loop diuretics on days 1 and 2 of intensive care unit (ICU) admission. In this study, a decrease in creatinine clearance (Cr CI) was observed in 24 patients compared to their baseline values. Numerous factors, naturally, can influence the decline in creatinine clearance in the intensive care setting, among them sepsis, medications, hypovolemia, blood pressure, and cardiac output. Patients with isolated cardiorenal syndrome, however, are frequently monitored in intensive care units, where loop diuretics are commonly prescribed. Our aim in writing this article is to provide further insights into the management of patients with cardiorenal syndrome admitted to intensive care, building on the findings reported by Kwok M. Ho et al. since such a finding could raise concerns in clinicians about the use of diuretics in the treatment of these patients [2]. In patients with decompensated heart failure, which may develop acutely upon admission to the intensive care unit or as a result of iatrogenic factors, creatinine clearance after loop diuretic administration may not only fall but, in some patients, rise instead. In which of these patients does CrCl fall and in which does it rise after a loop diuretic? By what mechanisms can CrCl decrease after loop diuretic administration? Based on the literature available to us, we would like to add briefly some mechanisms beyond those discussed by Kwok M. Ho et al. We therefore open the letter with a short account of the physiology underlying glomerular filtration regulation.

The maintenance of a constant glomerular filtration rate (GFR) despite fluctuations in blood pressure and intravascular volume is primarily governed by renal autoregulation, the tubuloglomerular feedback (TGF) mechanism, counter-regulatory hormones, and sympathetic nervous system [3]. After diuretic administration, reductions in estimated GFR (eGFR) and CrCl have been observed in healthy volunteers [4]. In the initial hours, marked natriuresis triggers neurohormonal activation, increasing proximal tubular sodium and water reabsorption. With continued administration of the same dose, a new steady state is established over several days in which sodium intake and excretion are balanced, an adaptive response referred to as the ‘diuretic braking’ phenomenon [3]. If the diuretic dose is increased or sodium and water intake is reduced, effective circulating volume declines, further amplifying neurohormonal activation. Then, excessive activation of the neurohormonal system may disrupt glomerular filtration regulation and thus GFR can fall, with progression to tubular ischemia in some cases [3].

Patients monitored in the ICU often have comorbid conditions such as diabetes mellitus, hypertension, and congestive heart failure. In these patients, renal autoregulatory capacity is usually impaired [5]. The presence of similar comorbidities among the participants in the study by Kwok M. Ho et al. raises the possibility that their renal autoregulatory capacity may have been reduced as well [1]. Natriuresis induced by furosemide activates the RAAS, and in the setting of reduced renal autoregulation, angiotensin II–mediated vasoconstriction may predominate, owing to attenuation of the myogenic response and reduced afferent arteriolar dilation. How the TGF mechanism responds to furosemide, on the other hand, remains only partly understood. Experimental animal models have reported that furosemide-induced inhibition of the NKCC (sodium–potassium–2 chloride cotransporter) at the apical membrane of the macula densa reduces intracellular chloride sensing. Falling chloride sensing then triggers PGE2-related renin release and raises Ang II levels, which produce afferent arteriolar vasoconstriction and can ultimately reduce CrCl [5]. In their study, Kwok M. Ho et al. propose that triggered TGF signaling caused afferent arteriolar vasoconstriction and contributed to the fall in CrCl [1]. Available data instead suggest that TGF signaling is not actually triggered after loop diuretic administration; reduced chloride sensing at the macula densa is thought to activate a prostaglandin-related renin pathway, which in turn produces afferent arteriolar vasoconstriction [6]. In summary, the factors considered relevant to afferent arteriolar vasoconstriction after a loop diuretic include heightened sympathetic activity, impaired renal autoregulation, and elevated RAAS activity. As a consequence, reduced renal blood flow and lower glomerular hydrostatic pressure are thought to drive the fall in CrCl. The decline in CrCl in patients of the study by Kwok M. Ho et al. likely arose, and probably mostly so, through these mechanisms. Further studies are needed on this point.

In patients admitted to the ICU due to acute decompensated heart failure (ADHF), while a negative fluid balance develops after furosemide given for decongestion, an increase in CrCl can be observed contrary to expectations. Including all heart failure patients in this category may not be the right course of action. Increases in eGFR and CrCl after furosemide have generally been observed in patients with right heart failure and elevated right atrial pressure (RAP), or in those with left heart failure in whom the RAP/PCWP (pulmonary capillary wedge pressure) ratio is increased [7]. Elevated right atrial pressure and right heart failure, by causing renal venous congestion and an increase in renal venous pressure, can reduce net filtration pressure, eGFR, and CrCl, which is termed congestive nephropathy [8]. Studies have found that ADHF patients presenting with elevated renal venous pressure and congestion had worse renal function at the time of hospital admission compared to other patients presenting with ADHF [9]. In this setting, it is thought that following decongestive therapy with diuretics or ultrafiltration, renal venous congestion and renal interstitial pressure can decrease, and presumably net intraglomerular filtration pressure increases. The increase in CrCl and eGFR after decongestive therapy in such patients has been explained by this mechanism [8,10]. The reduction in elevated neurohumoral system activity following decongestive therapy may also be influential in higher levels of CrCl and eGFR; a fall in neurohormonal activity was observed following decongestive therapy in patients presenting with NYHA class IV ADHF [11].

In a subset of patients presenting with ADHF, however, worsening renal function (WRF) has been noted after furosemide administration. Examination of the cardiac and hemodynamic features of these patients showed, at least on the data available to us, a diagnosis of diastolic heart failure or an ejection fraction above 40% [12,13]. Sub-analyses of the Evaluation Study of Congestive Heart Failure and Pulmonary Artery Catheterization Effectiveness (ESCAPE) trial showed that in patients without elevated RAP, eGFR and CrCl fell after furosemide administration [7]. In patients with systolic heart failure in whom CrCl fell after furosemide, on the other hand, the RAP/PCWP ratio was not elevated [7]. Such findings may help us anticipate that CrCl can fall after furosemide in patients admitted to the ICU with ADHF whose right heart function remains unaffected. Beyond the framework outlined above, intravascular congestion can develop in some patients through volume redistribution alone [14]. For example, in pulmonary edema following acute myocardial infarction or in pulmonary edema linked to a hypertensive episode, intravascular congestion may arise together with volume redistribution [14]. Such patients tend to obtain symptomatic relief with loop diuretics, and as intravascular decongestion is achieved, a decrease in CrCl can probably be observed. Even with such a fall in CrCl, mortality and morbidity do not appear to increase after decongestion [15]. Survival has probably been longer in patients who achieve decongestion than in those who do not [15–18]. Diuretic treatment should therefore be continued even at the expense of a rise in creatinine. Well-designed studies are needed on this subject.


Muammer Avcı Department of Nephrology, Isparta City Hospital, Republic of Turkey Ministry of Health, Isparta, Turkey glomerul07@gmail.com
Özgür Timuçin Kutlu Department of Internal Medicine, Isparta City Hospital, Republic of Turkey Ministry of Health, Isparta, Turkey


## Data Availability

No datasets were generated or analyzed during the current study.
